# Femtosecond time-resolved observation of butterfly vibration in electronically excited *o*-fluorophenol

**DOI:** 10.1038/s41598-017-14483-w

**Published:** 2017-11-10

**Authors:** Fengzi Ling, Shuai Li, Xinli Song, Yanmei Wang, Jinyou Long, Bing Zhang

**Affiliations:** 1 State Key Laboratory of Magnetic Resonance and Atomic and Molecular Physics, Wuhan Institute of Physics and Mathematics, Chinese Academy of Sciences, Wuhan, 430071 China; 2 University of Chinese Academy of Sciences, Beijing, 100049 China

## Abstract

The butterfly vibration during the hydrogen tunneling process in electronically excited *o*-fluorophenol has been visualized in real time by femtosecond time-resolved ion yield spectroscopy coupled with time-resolved photoelectron imaging technique. A coherent superposition of out-of-plane C–F butterfly motions is prepared in the first excited electronic state (S_1_). As the C–F bond vibrates with respect to the aromatic ring, the nuclear geometry varies periodically, leading to the corresponding variation in the photoionization channel. By virtue of the more favorable ionization probability from the nonplanar minimum via resonance with the Rydberg states, the evolution of the vibrational wave packet is manifested as a superimposed beat in the parent-ion transient. Moreover, time-resolved photoelectron spectra offer a direct mapping of the oscillating butterfly vibration between the planar geometry and nonplanar minimum. The beats for the photoelectron peaks originating from the planar geometry are out of phase with those from the nonplanar minimum. Our results provide a physically intuitive and complete picture of the oscillatory flow of energy responsible for the coherent vibrational motion on the excited state surface.

## Introduction

Ultrafast molecular vibration is one of the fundamental motions that characterize chemical bonding and decide reaction dynamics at the molecular level^1–3^. Over the past few decades, frequency-resolved spectroscopies such as infrared and Raman spectra^4^ and resonant two-photon ionization (R2PI) spectroscopy^5^ have devoted to providing detailed spectroscopic data about molecular vibration. However, due to the difficulties in preparing the well-defined superposition and probing effectively the nuclear wave-packet motion at the same time, the direct view of molecular vibration remains one of the most challenging problems in femtochemistry^6^. To visualize the evolving motion, the system must be excited on a timescale shorter than the vibrational period. In general, the timescale for molecular vibration ranges from less than 10^−13^ s to approximately 10^−14^ s. With the development of the laser techniques^6,7^, it has become attractive to follow the vibrational motions of isolated molecular systems in the time domain. Among multiple time-resolved methods, time-resolved photoionization spectroscopy, which involves a pump-probe framework, has offered rich and radical dynamical information about many elementary reactions in chemistry, physics, and biology^8–11^. After the molecule is prepared in the superposition state by coherent excitation of the eigenstates of interest with an initial laser pulse, the time-dependent vibrational motion is followed by monitoring either the parent-ion signal or the photoelectron kinetic energy (PKE) distribution^12–17^ as a function of the pump-probe delay time. If the probing transition is sensitive to the changes in interatomic distance, i.e., the time evolution of the vibrational wave packet, the measured signal will provide direct ‘snapshots’ of the molecular motions.

In recent years, several groups have employed such a technique to prepare a coherent vibrational wave packet and detect its dynamic evolution in several yet-cooled molecules^18–28^, including the early work by Reid and coworkers that observed obvious quantum beating patterns in the studies of *p*-difluorobenzene^18^, *p*-fluorotoluene^19,20^, and toluene^21,22^ using picosecond time-resolved photoelectron spectroscopy (TRPES). These beats arose from coupling between a small number of vibrational states and were a signature of restricted intramolecular vibrational energy redistribution (IVR). Later work by Suzuki *et al*. extended the study to the dynamical processes on the femtosecond time scale. They investigated the coherent phenomena in CS_2_^23,24^ and pyrazine^25,26^ using femtosecond time-resolved photoelectron imaging (TRPEI). More recently, by the careful selection of probe wavelengths combined with the intrinsic molecular properties, Stavros *et al*. and Ashfold *et al*. established the potential of time-resolved ion yield (TR-IY) spectroscopy for probing the vibrational wave packet dynamics in several aromatic species such as catechol^27^, syringol, and guaiacol^28^. However, to the best of our knowledge, there has been few experimental reports about the simultaneous application of femtosecond TR-IY and TRPEI to map the coherent molecular motion on the excited state surface.

In the present work, we open up a comprehensive experimental approach to visualize the excited-state molecular vibration correlated with evident structural changes in a model system *o*-fluorophenol. As one of the prototypes of intramolecular hydrogen-bonding systems, *o*-fluorophenol has been the subject of numerous spectroscopic studies, including excitation and dispersed laser-induced fluorescence spectroscopy^29^, two-color resonant two-photon ionization (2C-R2PI) spectroscopy^30,31^, hole burning and high resolution ultraviolet spectroscopy^32^, mass analyzed threshold ionization spectroscopy (MATI)^30^, Fourier transform-infrared (FT-IR) and FT-Raman spectroscopy^33^, infrared spectroscopy^34,35^, and femtosecond TRPEI^36^. A study by Oikawa *et al*. suggested that the transition from the ground electronic state (S_0_) to the first excited electronic state (S_1_) displayed two strong bands near the origin^29^. Afterwards, Meerts *et al*. concluded that the two bands arose from excitations to the zero and first overtone levels of the out-of-plane butterfly vibration (τ), respectively^32^. The τ mode corresponded to the inversion motion of the C–F bond with respect to the aromatic ring^32^. The objective of this study is to visualize this butterfly vibration by virtue of the structural changes caused by selective excitation of the overtone band τ_0_^2^. After a coherent superposition of the S_1_ origin and two quanta in τ is prepared by the pump pulse, femtosecond TR-IY and TRPEI are both applied to monitor the periodic variations in the ionization cross section and the PKE distribution during its periodic motion between different geometries.

## Results

### Geometric characterization

**Figure 1:**
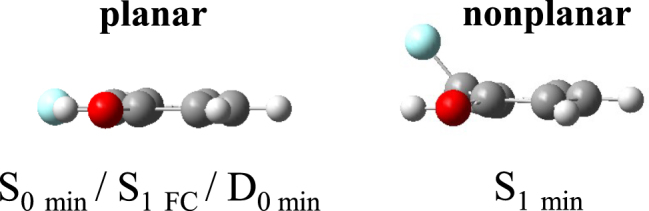
Schematics of nuclear geometries associated with the ground, excited, and ionic states in *o*-fluorophenol calculated at the theory level of TD-M052X with an aug-cc-pVDZ basis set.

Figure 1 shows the minimum-energy structures of the S_0_ state, the S_1_ state and the cationic ground state (D_0_) in *o*-fluorophenol. Although the S_0_ minimum geometry (S_0 min_) and the equilibrium geometry of the *o*-fluorophenol^+^ ion (D_0 min_) are both planar, the minimum energy geometry of the S_1_ state (S_1 min_) is nonplanar with rotation of the free F particle over 37° out of the aromatic plane. Immediately following excitation from the planar S_0 min_ geometry, S_1_ will be prepared in a planar geometry in the vertical Franck-Condon (FC) excitation region. Subsequently, the initially excited wave packet evolves away from the planar structure in the FC window towards the nonplanar S_1 min_ structure.

**Figure 2:**
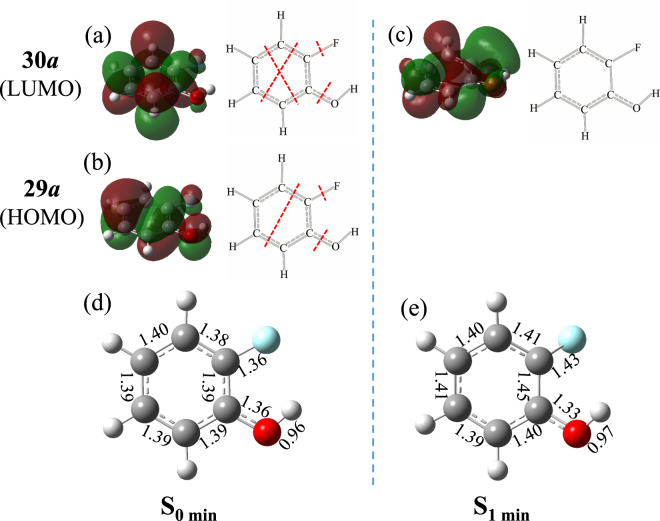
Nodal region patterns of the frontier orbitals in the minimum energy geometries of (**a**–**b**) S_0 min_ and (**c**) S_1 min_ calculated using TD-M052X/aug-cc-pVDZ.  Dash red lines are vertical nodal planes. Bond lengths (Å) are also indicated in (**d**) S_0 min_ and (**e**) S_1 min_.

To garner further insight into the origins of the different geometries in the minima of S_0_ and S_1_, we have also calculated the orbital energies and nodal properties of the highest occupied molecular orbital (HOMO) and the lowest unoccupied molecular orbital (LUMO). The energy gaps between HOMO and LUMO in S_0 min_ and S_1 min_ are determined to be 8.16 and 6.36 eV, respectively. As shown in Fig. 2 for the nodal patterns, the HOMO (29a) in S_0 min_ exhibits four nodal planes, three of which are perpendicular to the molecular plane and the other is parallel to the molecular plane. And the LUMO (30a) in S_0 min_ yields five nodal planes, four vertical planes and one horizontal plane. However, the 30a orbital in S_1 min_ shows only one horizontal nodal plane. It is obvious that the horizontal nodal plane is planar in S_0 min_ but distorted in S_1 min_. The S_1_ state of *o*-fluorophenol arises from electron promotion from a bonding π orbital of the oxygen atom to an antibonding π*orbital of the phenyl ring. In the FC region, the π* antibonding orbital is unstable and the ring expansion is expected, resulting in great changes on the molecular structure. The nonplanar equilibrium geometry agrees well with the smaller HOMO–LUMO gap and the distorted nodal structure in S_1 min_.

### Time-resolved ion yield measurements

**Figure 3:**
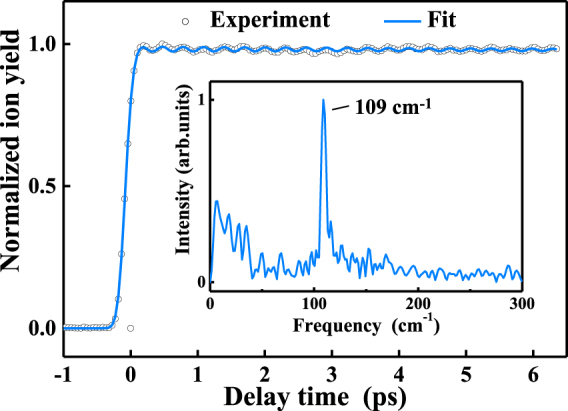
Transient recorded at the parent-ion channel following excitation at 271.3 nm and probing with 802 nm. The circles represent the experimental results, and the blue line is the fitting result. The inset shows the FFT of the residual given by subtracting the preliminary exponential fit from the experimental data.

Figure 3 presents the time-dependent ion signal of *o*-fluorophenol^+^ following excitation at 271.3 nm and probing with 802 nm. The molecules are excited close to the S_1_ origin with one-photon absorption at 271.3 nm, which are subsequently ionized by the absorption of at least three 802 nm photons. The experimental data is preliminarily fitted to a convolution of the cross-correlation function with an exponential decay, which yields a time constant of about ns scale. Due to the limited translation stage we used, the lifetime cannot be determined precisely enough. However, the long-lived lifetime is consistent with the results reported by Meerts’s group^32^ and Ashfold’s group^37^. Meerts’s group did high resolution fluorescence excitation experiments to estimate the lifetime of the S_1_ zero level to be 4.6 ns. Ashfold’s group reported a decay time of 2.3 ns by ultrafast time-resolved photoion spectroscopy when exciting at its S_1_–S_0_ origin (λ_pump_ = 272 nm) and ionizing at λ_probe_ = 243.1 nm. This long-lived behavior may arise by two mechanisms. One possibility is the IVR process. In light of the extremely low excess vibrational energy (i.e., less than 0.01 eV) in S_1_, we can exclude the possibility of IVR under our experimental conditions. The second possibility is O–H bond fission mediated via a hydrogen tunneling mechanism. The similar lifetime was also found in phenol^38^ and attributed to a tunneling process from the bound S_1_ (^1^ππ*) potential energy surface onto the dissociative S_2_ (^1^πσ*) surface. Therefore, it is reasonable to attribute the decay component with ns lifetime to the hydrogen tunneling time of the S_1_ state via an S_1_/S_2_ conical intersection.

Unlike the simple decay behavior of the parent-ion transient reported by Ashfold’s group^37^, a superimposed coherent oscillation due to a vibrational quantum beat appears in the decay profile. The beat frequency can be derived from a fast Fourier transform (FFT) of the residual obtained by subtracting the preliminary exponential fit from the experimental data. The FFT for the residual is displayed in the inset in the same figure and almost exclusively yields a single frequency with an associated energy centered at 109 cm^−1^, corresponding to a periodicity of 306 fs. To garner further insight into the mechanism of the oscillating signals, the frequency observed here is compared with the results of the frequency-resolved spectra of the *o*-fluorophenol S_1_ state^30,32^. Within our excitation pulse, the only FC active vibrational mode present is the first overtone of the out-of-plane butterfly vibration τ^2^. Specifically, the observed frequency at 109 cm^−1^ is in excellent agreement with the wave number separation between the S_1_ origin (S_1_, ν = 0) and two quanta vibrational progression of τ (S_1_, ν = 0, and τ_0_^2+^, respectively, ΔE = 109 cm^−1^)^32^. Hence, the superimposed quantum beat is the direct manifestation of the coherent excitation process and attributed to the variation in the ionization cross section as the vibrational wave packet moves back and forth from the planar FC region to the nonplanar minimum along τ. This is presumably due to a periodic change in the ionization mechanism, which will be discussed in more detail later combined with the associated time-dependent photoelectron images.

The transient can be modelled using a combination of (i) one exponential with the 1/e decay time of the non-oscillatory component τ_1_; this is convoluted with a Gaussian g(Δt) of width 203 fs to describe the laser cross-correlation (instrument response function) and (ii) an exponentially damped oscillation with frequency ν_osc_, associated phase-shift of φ, and damping time τ_damp_. The details of the fit procedure^27^ are described in following formula

1 $${\rm{I}}({\rm{\Delta }}t)=({\rm{g}}({\rm{\Delta }}t)\otimes {\rm{A}}\,{{\rm{e}}}^{\frac{-{\rm{\Delta }}t}{{\tau }_{{\rm{1}}}}})\times ([({\rm{B}}\,\cos ((2{{\rm{\pi }}{\rm{\nu }}}_{{\rm{osc}}}{\rm{\Delta }}t)+\phi )\times {{\rm{e}}}^{\frac{-{\rm{\Delta }}t}{{{\rm{\tau }}}_{{\rm{damp}}}}}]+{\rm{C}})$$

In the above, A–C describe the amplitude of each component function in the total fit. The colored line in Fig. 3 shows the fit to the corresponding transient. The best fit yields τ_damp_ = 8.3 ps for the damping constant and ν_osc_ = 109 cm^−1^ for the beat frequency, well in accord with the value obtained by the FFT of the residual. This consistency adds weight to our assignment of the beat to the coherent vibrational motion along the out-of-plane C–F butterfly vibration τ.

### Time-resolved photoelectron spectra

**Figure 4:**
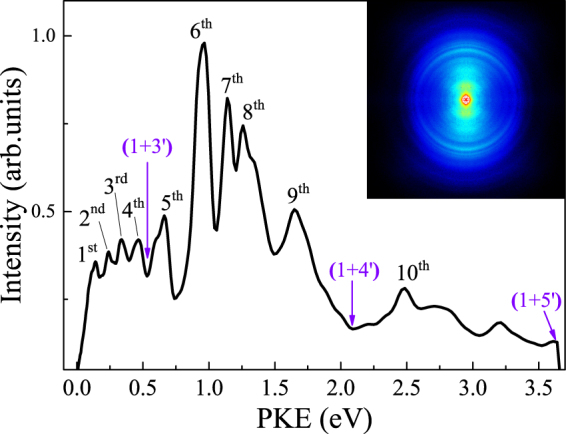
PKE distribution extracted from the image at Δt = 35 fs. The inset is the corresponding raw photoelectron image data at 35-fs pump-probe delay. The linear polarizations of the pump and probe lasers are both aligned vertically in the plane of the figure.

Figure 4 shows the PKE spectrum extracted from a pBASEX-inverted photoelectron image at Δt = 35 fs. The inset is the corresponding raw photoelectron image. The arrows indicate the maximum electron kinetic energy available in different ionization schemes, which can be given by

2 $${{\rm{E}}}_{{\rm{el}}}^{{\rm{\max }}}={{\rm{n}}}_{{\rm{1}}}{{\rm{h}}{\rm{\nu }}}_{271.3{\rm{nm}}}+{{\rm{n}}}_{2}{{\rm{h}}{\rm{\nu }}}_{802{\rm{nm}}}-{{\rm{IP}}}_{{\rm{ad}}}$$

where n_1_ and n_2_ are the photon numbers involved for 271.3 and 802 nm, respectively. IP_ad_ is the adiabatic ionization energy of *o*-fluorophenol^30^, 8.68 eV. And the maximum electron kinetic energies for the (1 + 3′), (1 + 4′), and (1 + 5′) ionization schemes are calculated to be 0.52, 2.07, and 3.61 eV, respectively. Ten peaks centered at 0.14, 0.24, 0.34, 0.46, 0.66, 0.97, 1.14, 1.26, 1.65, and 2.49 eV are observed in the PKE spectrum, and we assign them as the 1^st^, 2^nd^, 3^rd^, 4^th^, 5^th^, 6^th^, 7^th^, 8^th^, 9^th^, and 10^th^ peaks, respectively. It is noted that the energy spacing between the 9^th^ and 1^st^ peaks is 1.51 eV, close to the energy of one 802-nm photon, indicating that the 1^st^ and 9^th^ peaks are ascribed to the same origin differing by three and four photons at 802 nm. The similar case is also found in the 6^st^ and 10^th^ peaks. Therefore, we mainly focus on the first eight peaks in the following discussions.

**Figure 5:**
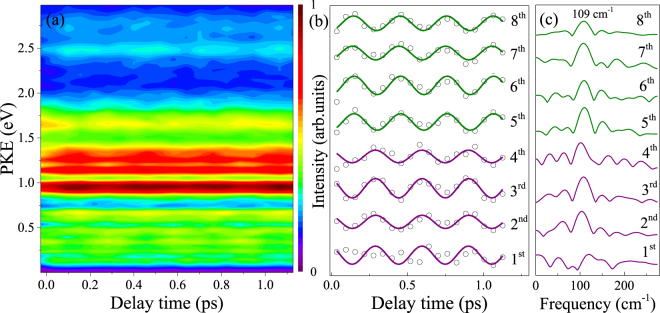
(**a**) Time-energy map of the PKE distribution. (**b**) Time evolutions of the energy selected photoelectron peak intensities. Experimental data points are given by the open circles, while sinusoidal fits are given by the solid lines. The purple and olive lines indicate the ionizations from the planar and nonplanar geometries, respectively. (**c**) Fourier transforms of the energy-resolved photoelectron signals for the eight peaks, yielding a frequency of 109 cm^−1^ corresponding to the butterfly vibration in S_1_.

In order to investigate the time-dependent behavior of the PKE distribution, a series of photoelectron images were measured at different time delays in steps of 60 fs. Figure 5(a) shows the time-energy map of the photoelectron intensities extracted from the measured images. The temporal profiles of the energy selected photoelectron peak intensities are extracted from Fig. 5(a) and presented as data points in Fig. 5(b). The transient behaviors of the photoelectron peaks are directly related to the variation of the molecular configuration in S_1_ from which ionization occurs. It is apparent that a superimposed coherent oscillation is observed in all the time dependences of the photoelectron peaks and well fitted by one sinusoidal function, plotted as solid lines in Fig. 5(b). The transforms of these oscillatory features, depicted in Fig. 5(c), exhibit one major frequency component at 109 cm^−1^. In accordance with the analysis of the parent-ion transient, the observed value agrees reasonably well with the overtone vibrational frequency of τ in S_1_. It is clear that the 1^st^, 2^nd^, 3^rd^, and 4^th^ peaks show a qualitatively similar changing trend in intensity with increasing pump-probe time delay. Namely, the quantum beats for the 1^st^, 2^nd^, 3^rd^, and 4^th^ peaks are in phase, indicating that they originate from the same geometry. In addition, the 5^th^, 6^th^, 7^th^, and 8^th^ peaks also show a similar changing trend in intensity with time, but their phase is different from that for the 1^st^, 2^nd^, 3^rd^, and 4^th^ peaks. This suggests that the 5^th^, 6^th^, 7^th^, and 8^th^ peaks are ionized from the other geometry. When the intensities of the 1^st^, 2^nd^, 3^rd^, and 4^th^ peaks are minimized, the intensities of 5^th^, 6^th^, 7^th^, and 8^th^ peaks are maximized, i.e., the beats originating from the two geometries are out of phase. These phase-shifted beats are the direct manifestation of the oscillatory flow of energy between different geometries. Since the nuclear geometry in the FC region of the S_1_ state is planar, the initial evolution of the prepared wave packet involves in the geometry rearrangement out of the planar geometry in the FC region towards the nonplanar minimum at a vibrational half-revival time. Therefore, the wave packet at time zero would mainly reflect the component character of the planar geometry, with the character of the nonplanar minimum growing in at later times. As seen in Fig. 5(b), a fast decay for the 1^st^, 2^nd^, 3^rd^, and 4^th^ peaks but a rapid growth for the 5^th^, 6^th^, 7^th^, and 8^th^ peaks are evident in the time-resolved PKE distributions recorded during the first 153 fs, indicating a fast energy transfer from the 1^st^, 2^nd^, 3^rd^, and 4^th^ peaks to the 5^th^, 6^th^, 7^th^, and 8^th^ peaks. The time of 153 fs corresponds to the evolution of the nuclear geometry away from a planar geometry in the vertical FC window towards the nonplanar S_1 min_ geometry. According to the above discussion, the 1^st^, 2^nd^, 3^rd^, and 4^th^ peaks are assigned to ionization from the planar geometry, but the 5^th^, 6^th^, 7^th^, and 8^th^ peaks are attributed to ionization from the nonplanar minimum.

## Discussion

According to the MATI spectra of *o*-fluorophenol recorded by ionizing via the 0^0^ and τ^2^ intermediate levels in the S_1_ state^30^, the most intense peaks correspond to those assigned as 0^+^, 1^1+^, and 8b^1+^ ion states with ion vibrational energies of 0, ~0.09, and ~0.20 eV respectively. One interesting issue is that the PKE for the 2^nd^ and 3^rd^ peaks are 0.12 and 0.22 eV lower in energy than that for the 4^th^ peak, respectively. Considering that the IP_ad_ has been determined to be 8.68 eV^30^, the photoelectron created by (1 + 3′) ionization process from the planar S_1 vFC_ geometry to the 0^+^ level of the D_0_ state will have the PKE around 0.52 eV, close to the center of the 4^th^ peak. Within the experimental error margins, it is credible that the 4^th^ peak is produced by the ionization from the planar S_1 vFC_ geometry to the 0^+^ ion state. Correspondingly, the 1^st^, 2^nd^, and 3^rd^ peaks are probably assigned to different ion vibrational states having good FC overlap with the planar geometry in the FC region in S_1_. The 2^nd^ and 3^rd^ peak correspond to the 8b^1+^ and 1^1+^ ion vibrational sates in D_0_, respectively. However, the specific assignment of the 1^st^ peak is not affirmative at present on account of lack of enough information. Upon close inspection of the 6^th^, 7^th^, and 8^th^ peaks, it is clear that they show a greatly enhanced intensity compared with the 1^st^, 2^nd^, 3^rd^, and 4^th^ peaks, suggesting that there may be intermediate states that affect the ionization process. Now we direct our attention to the 5^th^ peak, which may be caused by the following possible ionization mechanisms: (i) direct photoionization to D_0_; (ii) accidental resonance with the Rydberg state. Considering that the 5^th^ peak also originates from the ionization from the nonplanar minimum, the resonances with the Rydberg states for the 6^th^, 7^th^, and 8^th^ peaks will reduce greatly the possibility of the direct photoionization process for the 5^th^ peak. We therefore propose that the resonances with the Rydberg states are the most feasible origins for the 5^th^, 6^th^, 7^th^, and 8^th^ peaks. The PKE in the (1 + 2′) resonance-enhanced multiphoton ionization scheme via the resonance with the Rydberg state is described by^39^

3 $${\rm{PKE}}=2{{\rm{h}}{\rm{\nu }}}_{802}-\frac{{\rm{R}}}{{({\rm{n}}-{\rm{\delta }})}^{2}}$$

where R is the Rydberg constant, n is the principal quantum number, and δ is the quantum defect. According to Eq. (3), the δ values of the 5^th^, 6^th^, 7^th^, and 8^th^ peaks are calculated to be 0.64, 0.46, 0.35, and 0.27, respectively, with n of 3. For molecules composed of second-row atoms, typical δ values are 0.9–1.2 for s orbital, 0.3–0.5 for p orbital, and close to 0 for d orbital^40^. Therefore, the 5^th^ peak is assigned to the 3 s Rydberg state, while the 6^th^, 7^th^, and 8^th^ peaks are assigned to different magnetic sublevels of the 3p Rydberg states.

**Figure 6:**
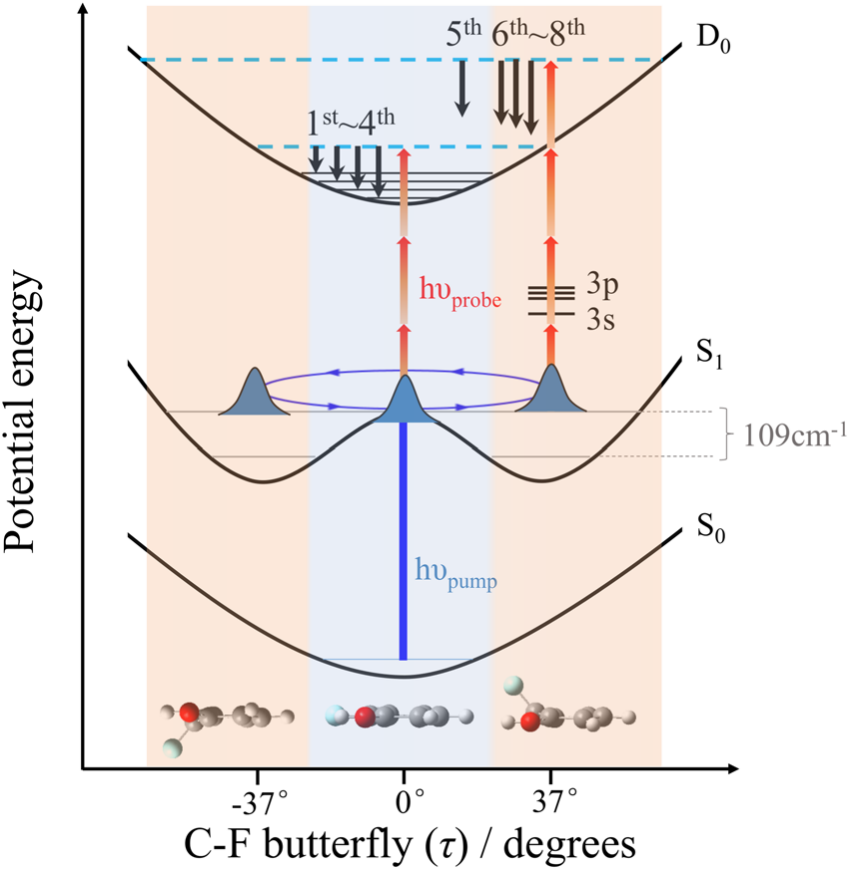
A schematic illustration of the ionization mechanism. The 1^st^, 2^nd^, 3^rd^, and 4^th^ peaks are generated from the planar geometry which are connected to different ion vibrational states. The 5^th^, 6^th^, 7^th^, and 8^th^ peaks are assigned to photoionization from the nonplanar minimum via the resonances with the 3 s and 3p Rydberg states.

Thus far, the whole mechanism for the possible ionization pathways during the early stages of the hydrogen tunneling process can be completely described, as shown in Fig. 6. The femtosecond pump pulse creates a vibrational wave packet by coherent excitation of the 0_0_^0^ and the τ_0_^2^ bands in S_1_, and subsequently, the ionizing probe laser pulse directly maps the coherent vibrational motion into the observed quantum beats in both the parent-ion transient and the time-dependent photoelectron intensities. Although similar beat patterns are also observed in several aromatic species^18–22,27,28^, the present work serves as the firstly experimental illustration about the simultaneous application of femtosecond TR-IY and TRPEI to map the excited-state molecular vibration under the same detection conditions. The butterfly motion can be described as a harmonic potential both in the S_0_ and D_0_ states but a double minimum potential in the S_1_ state along τ due to the equivalent minima at either side of the aromatic plane. At time zero, photoionization from the FC planar geometry occurs to different ion vibrational states in D_0_, producing the 1^st^, 2^nd^, 3^rd^, and 4^th^ peaks. After 153 fs, which corresponds to a half period of the C–F butterfly vibration, photoionization is induced from the nonplanar minimum. By the accidental resonances with different Rydberg states, the ionization efficiency from the nonplanar geometry is significantly enhanced, generating the 5^th^, 6^th^, 7^th^, and 8^th^ peaks. As the wave packet moves back and forth between the planar geometry and nonplanar minimum, variation of the nuclear geometries alters the photoionization channel, leading to the periodic changes in both the ionization cross section and the PKE distribution. The periodic variation in the ionization cross section with the geometry allows us to probe in real time the temporal evolution of the prepared vibrational wave packet, resulting in the superimposed beat in the parent-ion transient. More importantly, the sensitivity of the PKE distribution to the photoionization channel makes it possible to gain exquisite insight into the coherent vibrational motion between different geometries as the wave packet evolves on the excited state surface. The out-of-phase beats in the time-resolved PKE distributions are the direct manifestation of the periodically changing nuclear geometries, i.e. the C–F butterfly vibration.

In conclusion, the reported time domain experiments with femtosecond resolution give insights into the coherent vibrational motion in electronically excited *o*-fluorophenol. The coherent excitation of the out-of-plane C–F butterfly motions makes the nuclear geometry vary periodically. In the nonplanar minimum, the ionization from the S_1_ state is enhanced by the accidental resonance with different Rydberg states, thus modulating the ionization probability. A superimposed quantum beat is observed in parent-ion transient, possessing a frequency in accord with the progression in the τ mode. Furthermore, the sensitivity of the PKE distribution to the photoionization channel makes it possible to map directly the butterfly vibration between the planar geometry and nonplanar minimum. The time-dependent intensities of the photoelectron peaks originating from the planar geometry exhibit a clear beat with the similar periodicity but a phase shift of π rad with respect to the nonplanar minimum. Our results not only open up a promising route towards the real-time visualization of ultrafast molecular vibration in excited states, but also would be quite significant to provide insights into relevant studies of the manipulation of wavepacket motion (coherent control).

## Methods

### Experimental measurements

The experimental setup employed in the present work has been described in detail elsewhere^41,42^. Briefly, the seed pulse was generated by a self-mode-lock Ti:sapphire oscillator pumped by a continuous wave second harmonic of a Nd:YVO4 laser, and then amplified by a Nd:YLF pumped regenerative amplifier to deliver a 1 kHz pulse train centered at 802 nm of 100 fs duration with a maximum energy of 4.5 mJ/pulse. The femtosecond pump pulse at 271.3 nm centered around the S_1_ origin band^30^ (36800 cm^−1^), with a bandwidth of ~243 cm^−1^, was produced by an optical parametric amplifier (OPA) (Light Conversion, TOPAS-C). The 802 nm fundamental beam directly served as the probe light. To prevent one-color multiphoton processes, the two pulses were both softly focused into the interaction region by a fused silica lens of f = 400 mm with the typical energies of 0.1 μJ/pulse for the pump pulse and 32 μJ/pulse for the probe pulse, respectively. The polarizations of the laser pulses were individually controlled by Berek compensators (New Focus) and set to parallel to the face of the two-dimensional (2D) position sensitive detector. The probe beam was optically delayed with respect to the pump beam using a motorized translation stage (PI,M-126.CG1). The exact time-zero (∆t = 0) corresponding to temporal overlap of the laser pulses was measured by non-resonant multiphoton ionization of methanol. The Gaussian cross-correlation of the pump and probe laser pulses was also determined using the same method, yielding a value of 203 fs full width at half maximum (FWHM).

*o*-Fluorophenol (99% purity) in equilibrium with 2 bars of helium carrier gas expanded through a pulsed valve to form a supersonic expansion, which was skimmed and introduced into the ionization chamber where it intersected with the linear polarized laser beams perpendicularly. The resulting ions and photoelectrons were accelerated into a 36-cm field-free region by a set of electrostatic immersion lenses and then detected by a two-stage microchannel plate detector backed by a phosphor screen. The time-resolved transient of the parent ion was recorded by monitoring the current output directly from the phosphor screen using an oscilloscope. And the images on the screen were captured with a charge-coupled device camera. Each image was accumulated over 15 000 laser shots, and the backgrounds were removed by subtracting each beam image collected under the same conditions. The pBasex method^43^ was used to reconstruct the three-dimensional photoelectron speed distribution from the measured 2D image.

### Computational methodology

To gain further insight into the intrinsic molecular properties of *o*-fluorophenol, quantum calculations were performed to generate the minimum-energy structures of the S_0_, S_1_ and D_0_ states using the Gaussian 09 computational package^44^. The geometries were optimized with density function theory (DFT) for S_0_ and D_0_ but time dependent density function theory (TDDFT) for S_1_ at the M052X level of theory with the aug-cc-pVDZ basis set.
